# Role of Mast Cells and Type 2 Innate Lymphoid (ILC2) Cells in Lung Transplantation

**DOI:** 10.1155/2018/2785971

**Published:** 2018-10-30

**Authors:** Esmaeil Mortaz, Saeede Amani, Sharon Mumby, Ian M. Adcock, Mehrnaz Movassaghi, Jelle Folkerts, Johan Garssen, Gert Folkerts

**Affiliations:** ^1^Clinical Tuberculosis and Epidemiology Research Center, National Research Institute of Tuberculosis and Lung Diseases (NRITLD), Shahid Beheshti University of Medical Sciences, Tehran, Iran; ^2^Department of Immunology, School of Medicine, Shahid Beheshti University of Medical Sciences, Tehran, Iran; ^3^Cell and Molecular Biology Group, Airways Disease Section, National Heart and Lung Institute, Imperial College London, Dovehouse Street, London, UK; ^4^Priority Research Centre for Healthy Lungs, Hunter Medical Research Institute, The University of Newcastle, Newcastle, New South Wales, Australia; ^5^Division of Pharmacology, Utrecht Institute for Pharmaceutical Sciences, Faculty of Science, Utrecht University, Utrecht, Netherlands; ^6^Department of Pulmonary Medicine, Erasmus MC, Rotterdam, Netherlands; ^7^Nutricia Research, Immunology, Utrecht, Netherlands

## Abstract

The multifunctional role of mast cells (MCs) in the immune system is complex and has not fully been explored. MCs reside in tissues and mucous membranes such as the lung, digestive tract, and skin which are strategically located at interfaces with the external environment. These cells, therefore, will encounter external stimuli and pathogens. MCs modulate both the innate and the adaptive immune response in inflammatory disorders including transplantation. MCs can have pro- and anti-inflammatory functions, thereby regulating the outcome of lung transplantation through secretion of mediators that allow interaction with other cell types, particularly innate lymphoid cells (ILC2). ILC2 cells are a unique population of hematopoietic cells that coordinate the innate immune response against a variety of threats including infection, tissue damage, and homeostatic disruption. In addition, MCs can modulate alloreactive T cell responses or assist in T regulatory (Treg) cell activity. This paper outlines the current understanding of the role of MCs in lung transplantation, with a specific focus on their interaction with ILC2 cells within the engrafted organ.

## 1. Introduction

### 1.1. Mast Cells

The multifunctional role of mast cells (MCs) within the immune system has been clarified since their discovery by Paul Ehrlich in 1878 [1–3]. CD34^+^ progenitor cells circulate in the blood and migrate into peripheral tissues where they further differentiate into mature MCs under the influence of various tissue-specific factors such as extracellular matrix proteins, adhesion molecules, cytokines, and chemokines [4]. MCs act as key immune and inflammatory sentinels by initiating and shaping the inflammatory response through the rapid activation of IgE-dependent and -independent innate immune pathways [5–8]. The most well-known MC activation pathway involves IgE/Fc*ϵ*RI signaling, but MCs are also triggered via pattern recognition receptors such as Toll-like receptors (TLRs), complement, neuropeptides, cytokines, and many other stimuli [9].

MCs are present in all tissues and are particularly abundant in tissues and mucous membranes, such as the lung and digestive tract. MCs have this strategic later location in order to respond to external inflammatory stimuli and pathogens [4, 10, 11]. MCs can produce growth factors, costimulatory molecules, and numerous pro- and anti-inflammatory mediators. These cells are heterogeneous in nature [12], and the response to an external stressor may be altered by the local microenvironment. MCs release >200 mediators including prestored factors, such as histamine [2] and tryptase [13], as well as de novo synthesized such as chemokines and cytokines in response to allergic or nonimmune triggers [14, 15].

The important role of MCs in both the innate and adaptive immune responses [16–19] has led to speculation that MCs may play a crucial role in organ allograft rejection [17, 20–23]. In contrast to other immune-competent organs, the transplanted lung is constantly exposed to airborne antigens that may activate the local immune response and thereby modulate MC activity [10, 24–29]. For example, activated MCs release IL-2 [30], IL-7 [31], IL-3, IL-6, IL-9, IL-10, IFN-*γ*, and TNF-*α* and chemokines (CXCL8, CCL2, and CCL5) which have all been implicated in organ transplant and rejection [10, 14, 15, 25, 32]. In addition, MCs may enhance chronic rejection by the induction of fibrotic pathways [33] in the lung [29], kidney [34–36], and heart [37, 38].

Regulatory T cells (Tregs) are essential in maintaining tolerance to self-antigens, preventing excessive immune responses and in abrogating autoimmunity during graft rejection [39–41]. The use of MC-deficient mice has emphasized the important role of MCs in the activation of Treg-mediated immunoregulatory activities during transplant rejection [42]. In agreement with this, the absence of MCs is associated with significantly reduced cardiac allograft survival after heterotopic heart transplantation in rats [43]. Mechanistically, this may involve the ability of MCs to act as antigen-presenting cells and to mediate allograft reactions [12, 44].

Activated MCs influence the activity of many other cell types [45]. In turn, the function of MCs is controlled by factors such as proteases, complement [46], TLR ligands [47], and stem cell factor (SCF) released by other immune cells and by structural cells such as fibroblasts and smooth muscle cells. These factors either prime MCs for mediator release or directly induce MC degranulation [48].

MCs are histologically categorized into two phenotypes based on their protease content termed MC-tryptase (MCT) and MC-tryptase/chymase (MCTC) [24]. However, it remains unclear which MC phenotype is involved in regulating transplant rejection. The phenotype of MCs varies over time following transplantation with the MCTC being the main phenotype implicated in chronic rejection after fibrosis in the transplanted kidney [49]. Indeed, the phenotypic shift from MCT to MCTC cells may be associated with a progressive and potentially irreversible decline in allograft function [50].

These data together indicate that MCs are important immune effector cells during lung allograft rejection, but the role of these cells in organ transplant rejection is still not completely clear. Type 2 innate lymphoid cells (ILC2) cells are found in the vicinity of MCs in lung tissue, and both cell types can communicate with each other [51]. In addition, ILC2s are involved in epithelial and lung tissue repair [52, 53] and ILC2 are found in the lung parenchyma and bronchoalveolar lavage (BAL) fluid of subjects undergoing lung transplant [54]. In this review, we discuss how MCs and ILC2 can modulate transplant rejection of the lung.

### 1.2. Innate Lymphoid Cells (ILCs)

ILCs are a novel population of hematopoietic cells [55] that develop from common lymphoid progenitors in fetal liver and bone marrow [56, 57]. These cells are multifunctional and found throughout the body but are more prominent at barrier surfaces such as the lung and mucosal membranes [54, 58, 59]. Three types of ILCs exist (ILC1, 2, and 3), and these are functionally analogous to T-helper (Th) 1, Th2, and Th17 cell subsets [54, 60]. ILCs have a lymphoid morphology and release similar profiles of cytokines and eicosanoids as their respective Th cells but lack the T cell antigen receptor [60, 61]. Exposure of ILC progenitors (ILCP) to cytokines such as IL-25 and IL-33 induces ILC2 cells which are able to release cytokines IL-5, IL-9, and IL-13 [32, 54].

In the lung, ILC2s are mainly localized to the epithelium and perform a variety of protective immune functions [62, 63]. For example, ILC2s and their cytokines play critical roles in the protection of airway epithelial cells (ECs) against pathogens and regulate the repair of damaged cells [52, 64]. Since ILC2s have a protective role by organizing the innate immune response against infection and tissue damage, it is likely that they are involved in regulating transplant rejection [54, 65, 66].

**Figure 1:**
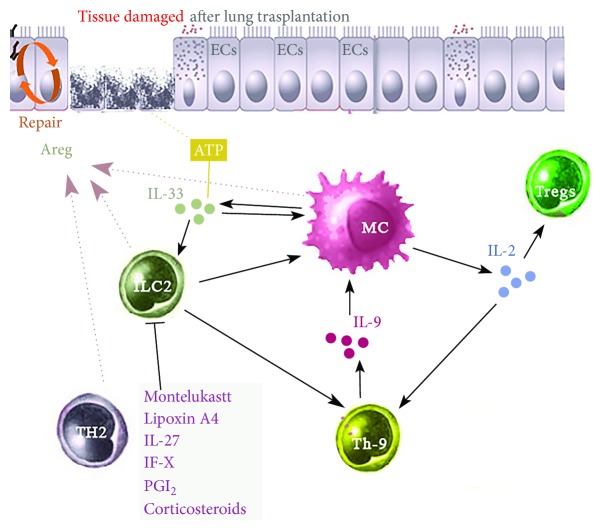
The role of Th9 cells and IL-9 in lung transplantation. IL-9 released by Th9 cells act on MCs to produce IL-2. MC-derived IL-2 leads to the expansion of CD45^+^ ILC2, which enhances the activation of Th9 cells and the further release of IL-9 in a feed-forward manner. In addition, IL-2 released from MCs induces tolerance in Treg cells and regulates transplantation survival. Activated ILC2s, MCs, and Th2 cells release Areg which is important in promoting EC repair from injury. Damaged epithelial cells, seen during transplant rejection, release IL-33 and ATP, which together can act on ILC2 and MCs to enhance their activity. A number of inhibitors including montelukast, IL-27, corticosteroid, PGI_2_, and lipoxin A4, can suppress the activation and/or proliferation of ILC2s and their release of inflammatory mediators. Abbreviations: Areg: amphiregulin; ECs: epithelial cells; IL: interleukin; ILC2: type 2 innate lymphoid cell; MCs: mast cells; PGI2: prostaglandin I2; Th9: T-helper type 9; Treg: T regulatory cells.

Expansion of ILC2s is driven by exposure to numerous immune factors including the cytokines IL-2 [67], IL-4 [68], IL-25, IL-33 [69–71], thymic stromal lymphopoietin (TSLP) [72], IL-9 [52, 73], IL-1*β* [69], and TNF-like ligand 1A [55]. In addition, eicosanoids such as prostaglandin D2 (PGD2) and leukotriene D4 (LTD4) [69] can drive the development of ILC2s. In contrast, inflammatory or immune suppressors such as montelukast, corticosteroids, prostaglandin L2, IL-27, IFN-*γ*, and lipoxin A4 suppress ILC2 proliferation and cytokine production [70, 74, 75] (Figure 1).

## 2. Role of MCs and ILC2s in Rejection/Survival of the Transplanted Lung

Since the first successful lung transplant in 1983 [76], the number of operations has grown substantially [77]. A lung transplant is generally the final treatment option for patients with end-stage lung disease. Various types of injury can damage a grafted organ. Some processes are due to the surgical procedure itself, for example, the sectioning of vessels and nerves or, in the transplanted lung, of the conducting airways. Other processes are inflammatory in nature, due to reperfusion of the graft or the onset of early allogeneic reactions. The lack of efficient tissue repair mechanisms could severely impair graft functioning, and the events involved in restoration of the transplanted airways utilize a variety of cell types [78].

### 2.1. The Role of IL-33 and IL-13

After lung transplant, IL-33 is released into the extracellular space which results in the activation of immune and inflammatory cells such as ECs, dendritic cells (DCs), MCs, ILC2s, and CD4^+^ T cells [79–82]. IL-33 is an alarm signal that triggers ECs in the lung and other cells present at the mucosal barrier, to reverse or prevent cell damage [20, 83, 84]. In addition, IL-33 recruits and activates cytokine production by ILC2s and by MCs [80, 82]. For example, ATP, released from the damaged epithelium and acting via the P2X7 receptor [66, 69, 83], in combination with IL-33, triggers the production of IL-13 by ILC2s. MCs can also release IL-33 following IgE cross-linking [51, 85].

IL-33, therefore, acts as a “sensor of cell injury” via MCs [86], and, in a feed-forward manner, activated MCs secrete additional IL-33 [20, 33, 85] which further enhances IL-13 release. IL-13 can prolong allograft survival associated with inhibition of IL-12 and TNF-*α* expression by DCs and macrophages. IL-13 does not directly activate T cells as they do not express the IL-13 receptor [87] indicating that the precise site of action of IL-13 requires further investigation. This is important since IL-13 is involved in transplant rejection as well [33] possibly through an effect on fibrosis [88]. IL-33 can expand and promote Tregs [80, 85, 89] and decrease the number of Th1 cells and their release of cytokines [80, 89]. Thus, IL-33 is implicated in the maintenance of allograft tolerance.

### 2.2. The Role of the IL-9/Th9 Nexus

IL-9 promotes the activation of both MCs [90] and ILC2s [91]. MCs produce IL-2 when activated by IL-9 released from IL-33-stimulated ILC2s. This MC-derived IL-2 release leads to expansion of proinflammatory CD25^+^ ILC2s and the release of cytokines from ILC2s which in turn activate Th9 cells [51]. Activated Th9 cells, in turn, release IL-9 which further enhances ILC2 and MC activation in the airways in a feed-forward manner [52]. In addition, IL-9 acts via MCs to induce tolerance in Tregs [42, 90] (Figure 1). IL-9 is likely to be important in lung rejection as anti-IL-9 treatment, at least in mice, reduced airway remodeling and TGF-*β*1 expression, and improved lung function in models of lung transplant rejection [92]. Importantly, there was a correlation between the reduction in MC numbers and decreased airway remodeling further indicating the important role of MCs in fibrosis [93]. This indicates that the IL-9/Th9 nexus can modulate transplant rejection by affecting the interaction between MCs and ILC2 [52].

### 2.3. Other Mediators

ILC2s express important immune molecules including GATA3, ROR*α*, BCL11B, EST1, G9a, and GFI1 [54, 69, 70]. In general, ILC2s respond rapidly to the presence of numerous factors involved in cell death via damage-associated molecular patterns (PAMPs) by releasing numerous cytokines that enable crosstalk with other immune cells [69]. Thus, MCs and ILC2s work coordinately to provide the optimal immune response needed to control survival or prevent rejection during transplant of the donor organ [91].

Human MCs produce lipid mediators such as PGD2 and LTD4 following Fc*ε*RI activation [31, 94–96]. PGD2 stimulates ILC2 migration into the lung and drives the production of type 2 cytokines via its receptor CRTH2 [97] which is a distinctive marker of human ILC2s [31, 98]. In contrast, IL-2 produced by ILC2s assists Treg survival [30, 99] and thus supports survival of the transplanted organ. In addition, activated lung ECs release PGD2 to enable recruitment of ILC2s, basophils, MCs, and Th2 cells into the inflamed airway [100].

ILC2s produce amphiregulin (Areg) which promotes epithelial cell repair [34, 101, 102]. Areg is also produced by MCs and Th2 cells which further indicates a critical feed-forward interaction within the MC-ILC2 nexus [91, 101, 103] (Figure 1). Conversely, ECs release factors such as IL-25, IL-33, and TSLP which drive ILC [91, 104]. IL-25 and IL-33 induce different types of ILCs. IL-33 induces ILC2s whereas IL-25 preferentially elicits multipotent progenitor- (MPP-) type 2 cells, a new population of innate cells which also promote type 2 immunity [105]. IL-7 has also been described as a crucial factor in the development of ILC2s [31]. The *in vivo* sources of IL-7 required for ILC development are unknown, but IL-7 is critical for the generation and maintenance of all lymphocytes and is expressed by stromal cells [106].

**Figure 2:**
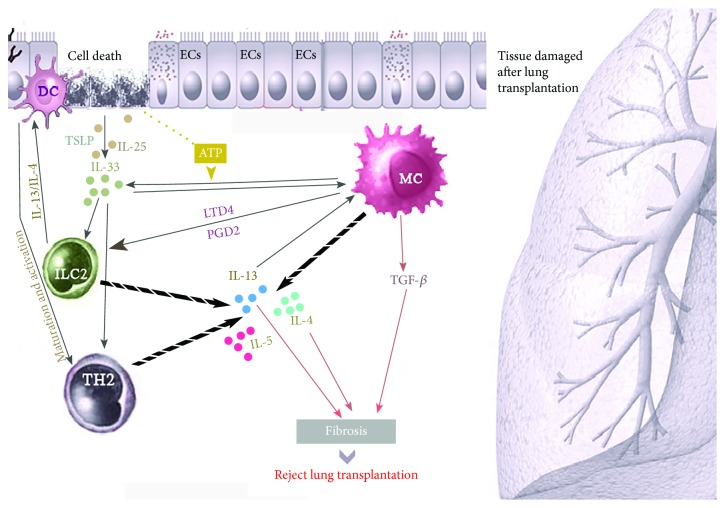
Interactions of mast cells (MC) and type 2 innate lymphoid cells (ILC2) in lung rejection after transplantation. After lung transplantation, damaged airway ECs release mediators such as IL-25, IL-33, and TSLP which caused activation of MCs and ILC2s and the subsequent release of IL-4, IL-5, and IL-13. IL-4 and IL-13 also enhance Th2 cell maturation and activation and stimulate DCs which interact with ECs to enhance the release of IL-33. MCs also produce IL-33 upon activation. The effect of IL-33 on ILC2-mediated release of IL-13 is enhanced by ATP produced by damaged ECs. LTD4 and PGD2 released by activated MCs further recruits and activates ILC2s to produce IL-2, −4, −5, −9, and −13. IL-13 released by Th2 cells plays a dual role in the induction of organ rejection and in promoting tolerance. Moreover, IL-13 can induce epithelial cell hyperproliferation and collagen deposition leading to pulmonary fibrosis. IL-4 released by MC also triggers fibroblast activation leading to lung fibrosis. MC-derived TGF-*β*1 further enhances fibrotic activity. Abbreviations: Areg: amphiregulin; DCs: dendritic cells; ECs: epithelial cell; IL: interleukin; LTD4: leukotriene D4; PGD2: prostaglandin D2; TGF-*β*1: transforming growth factor beta; Th2: T helper type 2 cell; TSLP: thymic stromal lymphopoietin.

These data highlight the interplay between MCs and ILC2s in maintaining the integrity of the respiratory epithelium and restoring the lung during infection of the transplanted lung [78, 107, 108] (Figure 2).

## 3. Possible Role of MCs and ILC2s in Lung Allograft Rejection

Due to the lung's distinctive anatomic position, long-term graft survival is comparatively lower than with other solid organs such as the heart, liver, and kidney [76]. According to the 2016 report, a half-life of heart, kidney, and liver transplants endures around 12 years but the median survival after lung transplantation in the same condition is around 5.8 years [109]. This may result from injury occurring during the lung transplant and the lack of organized tissue repair mechanisms which together damage the grafted organ [78] or an immunological reaction to the foreign organ leading to graft dysfunction and failure [110].

### 3.1. The Role of Fibrosis

Lung allograft rejection occurs due to both acute (AR) and chronic (CR) rejection processes [111–113]. Lung MCs play a dual role in the transplanted lung being implicated in both the induction of organ rejection and the induction of immune tolerance [10, 14, 50]. In contrast to the role of MC in AR, various studies have correlated CR with the fibrosis-inducing activity of MCs [10]. The proinflammatory cytokines TGF-*β*, IL-13, IL-1*β*, IL-17A, and IL-37 all contribute to the fibrotic [20]. IL-25 is also believed to be important as it induces a dramatic increase in both IL-13 and TGF-*β* expression in the lungs [70, 114].

Obliterative bronchiolitis (OB) occurs when graft survival is compromised after transplant of the donor lung [27, 113]. OB is the major cause of allograft rejection affecting at least 60% of recipients within 5 years of transplant [27, 115]. The release of profibrotic mediators such as TGF-*β*1 and basic fibroblast growth factor (bFGF) by MCs led to the examination of MCs in OB [116]. In addition to TGF-*β*1 and bFGF, other MC mediators including IL-4, TNF-*α*, histamine, heparin, chymase, and cathepsin G stimulate fibroblast proliferation and/or induce collagen synthesis [11, 31, 115, 117].

MCs and other immune cells accumulate around the vessels and airways during AR [118, 119]. Furthermore, MC hyperplasia occurs in areas of luminal fibrosis in both AR and CR of human lung allografts and is associated with the release of bFGF and histamine [29]. MC stabilization using cromolyn prevented the development of chronic lung allograft rejection in rats, again emphasizing the critical role of MCs in this process [95, 96]. Not only has bFGF been implicated in driving fibrosis-induced chronic lung allograft rejection but enhanced expression of bFGF may be a biomarker of rejection [37, 120]. Finally, TGF-*β*1 acts cooperatively with IL17 in fibrosis [115] and clinical observations indicate that TGF-*β*1 expression predicts the failure/success of the organ transplant [115, 121].

### 3.2. The Role of Th2 Cytokines

The cytokines IL-4 [10] and IL-13 [98], produced directly or indirectly by MCs and ILC2, are important during the development of chronic lung rejection [115]. IL-13 can drive transplant rejection due to fibrosis [31, 98]. ILC2-derived IL-13 promotes the migration of activated DCs into the local lymph nodes and thereby induce naïve T cells to differentiate into Th2 cells and increase IL-13 and IL-4 production [31, 122]. MC-derived IL-4 is an inducer of fibroblast activation during the development of chronic rejection [123].

In AR of lung allografts, MCs may increase allo-specific T cell responses which are unfavorable for the engrafted organ [22]. MCs activate T cells by presenting the antigen either directly in the context of MHC II [27, 124] or indirectly through the release of cytokines [10]. Numerous mediators produced by MCs and/or ILC2s [10, 31, 55, 60] have been implicated in either the survival or the rejection of transplanted lungs [125]. Many of these mediators act on T cells; for example, IL-4 and TNF-*α* can augment MHC-II on antigen-presenting cells (APC) and in the presence of IFN*α* induce T cell proliferation [116]. In addition to TNF-*α* inducing the recruitment of T cells and enhancing their interaction with antigen-presenting cells [10], MC-derived TNF-*α* can drive donor-derived DCs toward a tolerogenic phenotype [126]. Conversely, a reduction in the release of IL-10 and TGF-*β*1 from T cells supports the development of acute rejection [10] in part by effects on Tregs [127].

In summary, donor T cells are continually primed and activated to react against the host causing graft-versus-host disease (GvHD) that leads to tissue damage and death [40]. Finally, MC-derived mediators can upregulate the expression of adhesion molecules such as VCAM-1 and ICAM-1 on endothelial and granulocytes and enhance the trans-endothelial migration of T cells. This effect of MC products on adhesion molecule expression is amplified by crosstalk with ILC2s [52, 116].

## 4. Conclusion

ILC2s are juxtaposed with MCs in the lung and directly communicate with MCs to induce the release of numerous mediators from both cell types that are implicated in either the survival or the rejection of transplanted lungs. MCs affect ILC2 activities directly by the release of PGD2 or indirectly via the release of the proteases chymase and tryptase to promote IL-13 production and that of the other Th2 cytokines. IL-13 acts in a synergistic manner with IL-33 released from airway ECs to reverse or prevent tissue damage during the transplant. Moreover, IL-33 can directly activate mast cells to secrete additional IL-33 and further activate Th2 cytokine production in a feed-forward manner. IL-2 produced by ILC2s assists Treg survival and thus further supports survival of the transplanted organ.

In contrast, IL-2 produced by MCs leads to the expansion of CD25^+^ ILC2s that, in turn, stimulates the IL-9/Th9 nexus to induce tolerance in Treg cells and an immune environment that enables rejection. There is still much to be learnt about what determines the nature of MC-ILC2 interactions in distinct local settings during transplant rejection, and further investigations are required.

## References

[1] Beaven M. A. (2009). Our perception of the mast cell from Paul Ehrlich to now. *European Journal of Immunology*.

[2] Menzies F. M., Shepherd M. C., Nibbs R. J., Nelson S. M. (2011). The role of mast cells and their mediators in reproduction, pregnancy and labour. *Human Reproduction Update*.

[3] Metz M., Maurer M. (2007). Mast cells – key effector cells in immune responses. *Trends in Immunology*.

[4] Siebenhaar F., Redegeld F. A., Bischoff S. C., Gibbs B. F., Maurer M. (2018). Mast cells as drivers of disease and therapeutic targets. *Trends in Immunology*.

[5] Galli S. J., Nakae S., Tsai M. (2005). Mast cells in the development of adaptive immune responses. *Nature Immunology*.

[6] Galli S. J., Tsai M. (2012). IgE and mast cells in allergic disease. *Nature Medicine*.

[7] Heib V., Becker M., Taube C., Stassen M. (2008). Advances in the understanding of mast cell function. *British Journal of Haematology*.

[8] Marshall J. S. (2004). Mast-cell responses to pathogens. *Nature Reviews Immunology*.

[9] Yu Y., Blokhuis B. R., Garssen J., Redegeld F. A. (2016). Non-IgE mediated mast cell activation. *European Journal of Pharmacology*.

[10] Jungraithmayr W. (2015). The putative role of mast cells in lung transplantation. *American Journal of Transplantation*.

[11] Kitamura Y. (1989). Heterogeneity of mast cells and phenotypic change between subpopulations. *Annual Review of Immunology*.

[12] Suurmond J., van Heemst J., van Heiningen J. (2013). Communication between human mast cells and CD4^+^ T cells through antigen-dependent interactions. *European Journal of Immunology*.

[13] Vergnolle N., Wallace J. L., Bunnett N. W., Hollenberg M. D. (2001). Protease-activated receptors in inflammation, neuronal signaling and pain. *Trends in Pharmacological Sciences*.

[14] Erjefält J. S. (2014). Mast cells in human airways: the culprit?. *European Respiratory Review*.

[15] Moon T. C., Befus A. D., Kulka M. (2014). Mast cell mediators: their differential release and the secretory pathways involved. *Frontiers in Immunology*.

[16] Abraham S. N., St. John A. L. (2010). Mast cell-orchestrated immunity to pathogens. *Nature Reviews Immunology*.

[17] Bond G., Nowocin A., Sacks S. H., Wong W. (2015). Kinetics of mast cell migration during transplantation tolerance. *Transplant Immunology*.

[18] da Silva E. Z. M., Jamur M. C., Oliver C. (2014). Mast cell function: a new vision of an old cell. *Journal of Histochemistry & Cytochemistry*.

[19] Epelman S., Liu P. P., Mann D. L. (2015). Role of innate and adaptive immune mechanisms in cardiac injury and repair. *Nature Reviews Immunology*.

[20] Conti P., Caraffa A., Ronconi G. (2018). Mast cells participate in allograft rejection: can IL-37 play an inhibitory role?. *Inflammation Research*.

[21] Halloran P. F., Venner J. M., Madill-Thomsen K. S. (2018). Review: the transcripts associated with organ allograft rejection. *American Journal of Transplantation*.

[22] Hsiao H.-M., Scozzi D., Gauthier J. M., Kreisel D. (2017). Mechanisms of graft rejection after lung transplantation. *Current Opinion in Organ Transplantation*.

[23] Kritas S. K., Saggini A., Varvara G. (2013). Impact of Mast Cells in Rejection of Allografts. *European Journal of Inflammation*.

[24] Banga A., Budev M. M., Wang X., Hsieh F. (2015). Mast cell phenotypes in the allograft after lung transplantation. *The Journal of Heart and Lung Transplantation*.

[25] Barr M. L., Carey J. N., Nishanian G. P. (1998). Addition of a mast cell stabilizing compound to organ preservation solutions decreases lung reperfusion injury. *The Journal of Thoracic and Cardiovascular Surgery*.

[26] Greenland J. R., Xu X., Sayah D. M. (2014). Mast cells in a murine lung ischemia-reperfusion model of primary graft dysfunction. *Respiratory Research*.

[27] Martinu T., Chen D.-F., Palmer S. M. (2009). Acute rejection and humoral sensitization in lung transplant recipients. *Proceedings of the American Thoracic Society*.

[28] Spyridonidis A., Thomas A. K., Bertz H. (2004). Evidence for a graft-versus-mast-cell effect after allogeneic bone marrow transplantation. *Bone Marrow Transplantation*.

[29] Yousem S. A. (1997). The potential role of mast cells in lung allograft rejection. *Human Pathology*.

[30] Hershko A., Moshkovits I., Munitz A., Mekori Y. A., Salamon P. (2015). The mechanisms involved in IL-2 production by regulatory mast cells in chronic allergic dermatitis. *The Journal of Allergy and Clinical Immunology*.

[31] Ebbo M., Crinier A., Vély F., Vivier E. (2017). Innate lymphoid cells: major players in inflammatory diseases. *Nature Reviews Immunology*.

[32] Theoharides T. C., Valent P., Akin C. (2015). Mast cells, mastocytosis, and related disorders. *The New England Journal of Medicine*.

[33] Gieseck R. L., Wilson M. S., Wynn T. A. (2018). Type 2 immunity in tissue repair and fibrosis. *Nature Reviews Immunology*.

[34] Abo-Zenah H., Katsoudas S., Wild G. (2002). Early human renal allograft fibrosis: cellular mediators. *Nephron*.

[35] Goto E., Honjo S., Yamashita H., Shomori K., Adachi H., Ito H. (2002). Mast cells in human allografted kidney: correlation with interstitial fibrosis. *Clinical Transplantation*.

[36] Lajoie G., Nadasdy T., Laszik Z., Blick K. E., Silva F. G. (1996). Mast cells in acute cellular rejection of human renal allografts. *Modern Pathology*.

[37] Koskinen P. K., Kovanen P. T., Lindstedt K. A., Lemström K. B. (2001). Mast cells in acute and chronic rejection of rat cardiac allografts—a major source of basic fibroblast growth factor1. *Transplantation*.

[38] Li Q.-y., Raza-Ahmad A., MacAulay M. A. (1992). The relationship of mast cells and their secreted products to the volume of fibrosis in posttransplant hearts. *Transplantation*.

[39] McEwen S., Tang Q. (2017). Chapter 23-regulatory T cell therapy in transplantation. *Kidney Transplantation, Bioengineering and Regeneration*.

[40] Romano M., Tung S. L., Smyth L. A., Lombardi G. (2017). Treg therapy in transplantation: a general overview. *Transplant International*.

[41] Tang Q., Bluestone J. A. (2013). Regulatory T-cell therapy in transplantation: moving to the clinic. *Cold Spring Harbor Perspectives in Medicine*.

[42] Lu L.-F., Lind E. F., Gondek D. C. (2006). Mast cells are essential intermediaries in regulatory T-cell tolerance. *Nature*.

[43] Boerma M., Fiser W. P., Hoyt G. (2007). Influence of mast cells on outcome after heterotopic cardiac transplantation in rats. *Transplant International*.

[44] Gaudenzio N., Laurent C., Valitutti S., Espinosa E. (2013). Human mast cells drive memory CD4^+^ T cells toward an inflammatory IL-22^+^ phenotype. *The Journal of Allergy and Clinical Immunology*.

[45] Molderings G. J., Haenisch B., Brettner S. (2016). Pharmacological treatment options for *mast cell activation disease*. *Naunyn-Schmiedeberg's Archives of Pharmacology*.

[46] Marc M. M., Korosec P., Kosnik M. (2004). Complement factors c3a, c4a, and c5a in chronic obstructive pulmonary disease and asthma. *American Journal of Respiratory Cell and Molecular Biology*.

[47] Kulka M., Metcalfe D. D. (2006). TLR3 activation inhibits human mast cell attachment to fibronectin and vitronectin. *Molecular Immunology*.

[48] Columbo M., Horowitz E. M., Botana L. M. (1992). The human recombinant c-kit receptor ligand, rhSCF, induces mediator release from human cutaneous mast cells and enhances IgE-dependent mediator release from both skin mast cells and peripheral blood basophils. *The Journal of Immunology*.

[49] Yamada M., Ueda M., Naruko T. (2001). Mast cell chymase expression and mast cell phenotypes in human rejected kidneys. *Kidney International*.

[50] Banga A., Han Y., Wang X., Hsieh F. H. (2016). Mast cell phenotypes in the allograft after lung transplantation. *Clinical Transplantation*.

[51] Symowski C., Voehringer D. (2017). Interactions between innate lymphoid cells and cells of the innate and adaptive immune system. *Frontiers in Immunology*.

[52] Moretti S., Renga G., Oikonomou V. (2017). A mast cell-ILC2-Th9 pathway promotes lung inflammation in cystic fibrosis. *Nature Communications*.

[53] Vacca P., Montaldo E., Croxatto D. (2016). NK cells and other innate lymphoid cells in hematopoietic stem cell transplantation. *Frontiers in Immunology*.

[54] Marashian S. M., Mortaz E., Jamaati H. R. (2015). Role of innate lymphoid cells in lung disease. *Iranian Journal of Allergy, Asthma and Immunology*.

[55] Artis D., Spits H. (2015). The biology of innate lymphoid cells. *Nature*.

[56] Constantinides M. G., McDonald B. D., Verhoef P. A., Bendelac A. (2014). A committed precursor to innate lymphoid cells. *Nature*.

[57] Klose C. S. N., Flach M., Möhle L. (2014). Differentiation of type 1 ILCs from a common progenitor to all helper-like innate lymphoid cell lineages. *Cell*.

[58] Monticelli L. A., Sonnenberg G. F., Artis D. (2012). Innate lymphoid cells: critical regulators of allergic inflammation and tissue repair in the lung. *Current Opinion in Immunology*.

[59] Spits H., Di Santo J. P. (2011). The expanding family of innate lymphoid cells: regulators and effectors of immunity and tissue remodeling. *Nature Immunology*.

[60] Spits H., Artis D., Colonna M. (2013). Innate lymphoid cells — a proposal for uniform nomenclature. *Nature Reviews Immunology*.

[61] Vély F., Barlogis V., Vallentin B. (2016). Evidence of innate lymphoid cell redundancy in humans. *Nature Immunology*.

[62] Lund S., Walford H. H., Doherty T. A. (2013). Type 2 innate lymphoid cells in allergic disease. *Current Immunology Reviews*.

[63] Roediger B., Weninger W. (2015). Group 2 innate lymphoid cells in the regulation of immune responses. *Advances in Immunology*.

[64] Monticelli L. A., Sonnenberg G. F., Abt M. C. (2011). Innate lymphoid cells promote lung-tissue homeostasis after infection with influenza virus. *Nature Immunology*.

[65] Fukushi M., Ito T., Oka T. (2011). Serial histopathological examination of the lungs of mice infected with influenza a virus PR8 strain. *PLoS One*.

[66] Shimokawa C., Kanaya T., Hachisuka M. (2017). Mast cells are crucial for induction of group 2 innate lymphoid cells and clearance of helminth infections. *Immunity*.

[67] Oliphant C. J., Hwang Y. Y., Walker J. A. (2014). MHCII-mediated dialog between group 2 innate lymphoid cells and CD4^+^ T cells potentiates type 2 immunity and promotes parasitic helminth expulsion. *Immunity*.

[68] Motomura Y., Morita H., Moro K. (2014). Basophil-derived interleukin-4 controls the function of natural helper cells, a member of ILC2s, in lung inflammation. *Immunity*.

[69] Morita H., Moro K., Koyasu S. (2016). Innate lymphoid cells in allergic and nonallergic inflammation. *The Journal of Allergy and Clinical Immunology*.

[70] Cheng H., Jin C., Wu J., Zhu S., Liu Y.-J., Chen J. (2017). Guards at the gate: physiological and pathological roles of tissue-resident innate lymphoid cells in the lung. *Protein & Cell*.

[71] Kim B. S., Siracusa M. C., Saenz S. A. (2013). TSLP Elicits IL-33–Independent Innate Lymphoid Cell Responses to Promote Skin Inflammation. *Science Translational Medicine*.

[72] Mjösberg J., Bernink J., Golebski K. (2012). The transcription factor GATA3 is essential for the function of human type 2 innate lymphoid cells. *Immunity*.

[73] Turner J.-E., Morrison P. J., Wilhelm C. (2013). IL-9–mediated survival of type 2 innate lymphoid cells promotes damage control in helminth-induced lung inflammation. *Journal of Experimental Medicine*.

[74] Kortekaas Krohn I., Shikhagaie M. M., Golebski K. (2018). Emerging roles of innate lymphoid cells in inflammatory diseases: clinical implications. *Allergy*.

[75] Mchedlidze T., Kindermann M., Neves A. T., Voehringer D., Neurath M. F., Wirtz S. (2016). IL-27 suppresses type 2 immune responses in vivo via direct effects on group 2 innate lymphoid cells. *Mucosal Immunology*.

[76] Chang J.-C. (2013). *Gastric fluid aspiration-mediated pulmonary allograft failure, [Ph.D. thesis]*.

[77] Christie J. D., Edwards L. B., Kucheryavaya A. Y. (2012). The registry of the International Society for Heart and Lung Transplantation: 29th adult lung and heart-lung transplant report—2012. *The Journal of Heart and Lung Transplantation*.

[78] Da Silva C. A., Adda M., Stern M., de Blay F., Frossard N., Israel-Biet D. (2006). Marked stem cell factor expression in the airways of lung transplant recipients. *Respiratory Research*.

[79] Brown M. A., Weinberg R. B. (2018). Mast cells and innate lymphoid cells: underappreciated players in CNS autoimmune demyelinating disease. *Frontiers in Immunology*.

[80] Gajardo Carrasco T., Morales R. A., Pérez F. (2015). *Alarmin’* immunologists: IL-33 as a putative target for modulating T cell-dependent responses. *Frontiers in Immunology*.

[81] Scott I. C., Houslay K. F., Cohen S. (2016). Prospects to translate the biology of IL-33 and ST2 during organ transplantation into therapeutics to treat graft-versus-host disease. *Annals of Translational Medicine*.

[82] Toki S., Goleniewska K., Reiss S. (2018). Glucagon-like peptide 1 signaling inhibits allergen-induced lung IL-33 release and reduces group 2 innate lymphoid cell cytokine production in vivo. *The Journal of Allergy and Clinical Immunology*.

[83] Cayrol C., Girard J.-P. (2014). IL-33: an alarmin cytokine with crucial roles in innate immunity, inflammation and allergy. *Current Opinion in Immunology*.

[84] Saluja R., Khan M., Church M. K., Maurer M. (2015). The role of IL-33 and mast cells in allergy and inflammation. *Clinical and Translational Allergy*.

[85] Turnquist H. R., Zhao Z., Rosborough B. R. (2011). IL-33 expands suppressive CD11b^+^ Gr-1^int^ and regulatory T cells, including ST2L^+^ Foxp3^+^ cells, and mediates regulatory T cell-dependent promotion of cardiac allograft survival. *The Journal of Immunology*.

[86] Enoksson M., Lyberg K., Möller-Westerberg C., Fallon P. G., Nilsson G., Lunderius-Andersson C. (2011). Mast cells as sensors of cell injury through IL-33 recognition. *The Journal of Immunology*.

[87] Davidson C., Verma N. D., Robinson C. M. (2007). IL-13 prolongs allograft survival: association with inhibition of macrophage cytokine activation. *Transplant Immunology*.

[88] Wynn T. A., Ramalingam T. R. (2012). Mechanisms of fibrosis: therapeutic translation for fibrotic disease. *Nature Medicine*.

[89] Dai C., Lu F.-N., Jin N. (2016). Recombinant IL-33 prolongs leflunomide-mediated graft survival by reducing IFN-*γ* and expanding CD4^+^Foxp3^+^ T cells in concordant heart transplantation. *Laboratory Investigation*.

[90] Feng L.-L., Gao J.-M., Li P.-P., Wang X. (2011). IL-9 contributes to immunosuppression mediated by regulatory T cells and mast cells in B-cell non-Hodgkin’s lymphoma. *Journal of Clinical Immunology*.

[91] Hammad H., Lambrecht B. N. (2015). Barrier epithelial cells and the control of type 2 immunity. *Immunity*.

[92] McMillan S. J., Xanthou G., Lloyd C. M. (2005). Manipulation of allergen-induced airway remodeling by treatment with anti-TGF-*β* antibody: effect on the Smad signaling pathway. *The Journal of Immunology*.

[93] Brown J. M., Swindle E. J., Kushnir-Sukhov N. M., Holian A., Metcalfe D. D. (2007). Silica-directed mast cell activation is enhanced by scavenger receptors. *American Journal of Respiratory Cell and Molecular Biology*.

[94] Barnig C., Cernadas M., Dutile S. (2013). Lipoxin A_4_ regulates natural killer cell and type 2 innate lymphoid cell activation in asthma. *Science Translational Medicine*.

[95] Chang J. E., Doherty T. A., Baum R., Broide D. (2014). Prostaglandin D2 regulates human type 2 innate lymphoid cell chemotaxis. *The Journal of Allergy and Clinical Immunology*.

[96] Chang J.-C., Leung J., Tang T. (2014). Cromolyn ameliorates acute and chronic injury in a rat lung transplant model. *The Journal of Heart and Lung Transplantation*.

[97] Voehringer D. (2013). Protective and pathological roles of mast cells and basophils. *Nature Reviews Immunology*.

[98] Campillo-Navarro M., Chavez-Blanco A., Wong-Baeza I. (2014). Mast cells in lung homeostasis: beyond type I hypersensitivity. *Current Respiratory Medicine Reviews*.

[99] Boyman O., Sprent J. (2012). The role of interleukin-2 during homeostasis and activation of the immune system. *Nature Reviews Immunology*.

[100] Xue L., Salimi M., Panse I. (2014). Prostaglandin D_2_ activates group 2 innate lymphoid cells through chemoattractant receptor-homologous molecule expressed on T_H_2 cells. *The Journal of Allergy and Clinical Immunology*.

[101] Ogata-Suetsugu S., Yanagihara T., Hamada N. (2017). Amphiregulin suppresses epithelial cell apoptosis in lipopolysaccharide-induced lung injury in mice. *Biochemical and Biophysical Research Communications*.

[102] Zaiss D. M. W., Gause W. C., Osborne L. C., Artis D. (2015). Emerging functions of amphiregulin in orchestrating immunity, inflammation, and tissue repair. *Immunity*.

[103] de Kleer I. M., Kool M., de Bruijn M. J. W. (2016). Perinatal activation of the interleukin-33 pathway promotes type 2 immunity in the developing lung. *Immunity*.

[104] Saenz S. A., Siracusa M. C., Perrigoue J. G. (2010). IL25 elicits a multipotent progenitor cell population that promotes T_H_2 cytokine responses. *Nature*.

[105] Saenz S. A., Siracusa M. C., Monticelli L. A. (2013). IL-25 simultaneously elicits distinct populations of innate lymphoid cells and multipotent progenitor type 2 (MPP^type2^) cells. *Journal of Experimental Medicine*.

[106] McKenzie A. N. J., Spits H., Eberl G. (2014). Innate lymphoid cells in inflammation and immunity. *Immunity*.

[107] Krause D. S., Theise N. D., Collector M. I. (2001). Multi-organ, multi-lineage engraftment by a single bone marrow-derived stem cell. *Cell*.

[108] Yamada M., Kubo H., Kobayashi S. (2004). Bone marrow-derived progenitor cells are important for lung repair after lipopolysaccharide-induced lung injury. *The Journal of Immunology*.

[109] Thabut G., Mal H. (2017). Outcomes after lung transplantation. *Journal of Thoracic Disease*.

[110] Nakano T., Lai C.-Y., Goto S. (2012). Immunological and regenerative aspects of hepatic mast cells in liver allograft rejection and tolerance. *PLoS One*.

[111] Martinu T., Pavlisko E. N., Chen D.-F., Palmer S. M. (2011). Acute allograft rejection: cellular and humoral processes. *Clinics in Chest Medicine*.

[112] Sato M., Waddell T. K., Wagnetz U. (2011). Restrictive allograft syndrome (RAS): a novel form of chronic lung allograft dysfunction. *The Journal of Heart and Lung Transplantation*.

[113] Verleden G. M., Raghu G., Meyer K. C., Glanville A. R., Corris P. (2014). A new classification system for chronic lung allograft dysfunction. *The Journal of Heart and Lung Transplantation*.

[114] Hams E., Armstrong M. E., Barlow J. L. (2014). IL-25 and type 2 innate lymphoid cells induce pulmonary fibrosis. *Proceedings of the National Academy of Sciences of the United States of America*.

[115] Iwashima M., Love R. (2013). Potential of targeting TGF-*β* for organ transplant patients. *Future Medicinal Chemistry*.

[116] Strieter R. M. (2005). Pathogenesis and natural history of usual interstitial pneumonia: the whole story or the last chapter of a long novel. *Chest*.

[117] Feghali C. A., Bost K. L., Boulware D. W., Levy L. S. (1992). Human recombinant interleukin-4 induces proliferation and interleukin-6 production by cultured human skin fibroblasts. *Clinical Immunology and Immunopathology*.

[118] Itoh S., Nakae S., Velotta J. B. (2010). The role of recipient mast cells in acute and chronic cardiac allograft rejection in C57BL/6-*Kit^W-sh/W-sh^* mice. *The Journal of Heart and Lung Transplantation*.

[119] O'Keeffe C., Baird A. W., Nolan N., McCormick P. (2002). Mast cell hyperplasia in chronic rejection after liver transplantation. *Liver Transplantation*.

[120] Lee A. G. L., Wagner F. M., Giaid A. (1997). Immunohistochemical characterization of inflammatory and proliferative events during chronic rejection in rat lung allografts1. *Transplantation*.

[121] Brunet M. (2012). Cytokines as predictive biomarkers of alloreactivity. *Clinica Chimica Acta*.

[122] von Moltke J., Pepper M. (2018). Sentinels of the type 2 immune response. *Trends in Immunology*.

[123] Walgenbach K. J., Heeckt P. F., Stanson J. D., Whiteside T. L., Bauer A. J. (1996). Increased presence of mast cells and interleukin-4 during chronic rejection of rat intestinal allografts. *Transplantation Proceedings*.

[124] Nakae S., Suto H., Iikura M. (2006). Mast cells enhance T cell activation: importance of mast cell costimulatory molecules and secreted TNF. *The Journal of Immunology*.

[125] Moiseeva E. P., Bradding P. (2011). Mast cells in lung inflammation. *Mast Cell Biology*.

[126] de Vries V. C., Pino-Lagos K., Nowak E. C., Bennett K. A., Oliva C., Noelle R. J. (2011). Mast cells condition dendritic cells to mediate allograft tolerance. *Immunity*.

[127] Depinay N., Hacini F., Beghdadi W., Peronet R., Mécheri S. (2006). Mast cell-dependent down-regulation of antigen-specific immune responses by mosquito bites. *The Journal of Immunology*.

